# Complex offspring size effects: variations across life stages and between species

**DOI:** 10.1002/ece3.1320

**Published:** 2015-02-14

**Authors:** Zhao Sun, Jean-François Hamel, Christopher C Parrish, Annie Mercier

**Affiliations:** 1 Department of Ocean Sciences, Memorial UniversitySt. John's, Newfoundland and Labrador, A1C 5S7, Canada; 2 Society for the Exploration and Valuing of the Environment (SEVE)21 Phils Hill Road, Portugal Cove-St. Philips, Newfoundland and Labrador, A1M 2B7, Canada

**Keywords:** Benthic environment, carry-over effect, cnidarian, life history, offspring performance, parental care, size–number trade-off, viviparity

## Abstract

Classical optimality models of offspring size and number assume a monotonically increasing relationship between offspring size and performance. In aquatic organisms with complex life cycles, the size–performance function is particularly hard to grasp because measures of performance are varied and their relationships with size may not be consistent throughout early ontogeny. Here, we examine size effects in premetamorphic (larval) and postmetamorphic (juvenile) stages of brooding marine animals and show that they vary contextually in strength and direction during ontogeny and among species. Larger offspring of the sea anemone *Urticina felina* generally outperformed small siblings at the larval stage (i.e., greater settlement and survival rates under suboptimal conditions). However, results differed when analyses were conducted at the intrabrood versus across-brood levels, suggesting that the relationship between larval size and performance is mediated by parentage. At the juvenile stage (15 months), small offspring were less susceptible than large ones to predation by subadult nudibranchs and both sizes performed similarly when facing adult nudibranchs. In a sympatric species with a different life history (*Aulactinia stella*), all juveniles suffered similar predation rates by subadult nudibranchs, but smaller juveniles performed better (lower mortalities) when facing adult nudibranchs. Size differences in premetamorphic performance of *U. felina* were linked to total lipid contents of larvae, whereas size-specific predation of juvenile stages followed the general predictions of the optimal foraging strategy. These findings emphasize the challenge in gathering empirical support for a positive monotonic size–performance function in taxa that exhibit complex life cycles, which are dominant in the sea.

## Introduction

A key principle of life-history theory is the occurrence of a trade-off between the number and size of offspring produced (Smith and Fretwell 1974; Stearns 1992). This trade-off is driven by the balance between energy spent on individual offspring and parental fitness (Smith and Fretwell 1974), with two important underlying assumptions: (1) a negative relationship between offspring number and energy invested per offspring and (2) a positive relationship between investment per offspring and offspring performance (quality). Both assumptions have been explored from numerous angles, in countless taxa and environments, with variable outcomes. In aquatic systems, some studies have determined that offspring (egg) size reflects parental investment (Quattro and Weeks 1991; Jaeckle 1995) and the amount of reserves available for early growth, while others have shown that energetic content and egg size are not always directly related (Moran et al. 2013). Contrary to terrestrial models such as insects and birds (Fox and Czesak 2000; Krist 2011), support for the assumption that larger offspring perform better has been inconsistent in aquatic models (Sogard 1997; Moran 1999; Marshall et al. 2003; Dziminski and Roberts 2006; Dibattista et al. 2007). It has been proposed that the relationship between offspring size and performance may not always be monotonic (Hendry et al. 2001).

In animals with complex (biphasic or benthopelagic) life cycles, which dominate the faunal diversity in the ocean (and overall), size is suggested to influence the premetamorphic performance of offspring, for example, fertilization success (Levitan 2000, 2006). In addition, larger larvae have been shown to display a greater ability to delay settlement in the absence of proper settlement cues (Marshall and Keough 2003). Offspring size may also influence postmetamorphic performance, including survival, growth, competition among conspecifics, and reproduction of the next generation (Emlet and Hoegh-Guldberg 1997; Emlet and Sadro 2006). Current studies of size-related offspring performance in benthic organisms have most often focused on a single life stage (especially the postmetamorphic stage), whereas limited empirical data exist on size-related fitness across multiple life stages (Moran and Emlet 2001; Marshall et al. 2006; Rius et al. 2009; Dias and Marshall 2010; Allen and Marshall 2014). In addition, investigations in this field have centered on ascidians and bryozoans (e.g., Marshall and Keough 2005, 2008; Dias and Marshall 2010; Jacobs and Sherrard 2010), and a few species of echinoderms, molluscs, crustaceans, and annelids (e.g., Emlet and Hoegh-Guldberg 1997; Moran and Emlet 2001; Emlet and Sadro 2006; Allen and Marshall 2014). Other phyla and species from outside tropical or warm temperate regions are underrepresented.

While it is commonly assumed that size confers advantages, contrasting results have been reported in aquatic systems (e.g., Marshall and Keough 2005 vs. Jacobs and Sherrard 2010). The influence of offspring size on their performance appears to be strongly mediated by external conditions, including predation (Rivest 1983; Barbeau and Scheibling 1994; Bell et al. 2011), competition (Marshall et al. 2006; Allen et al. 2008), temperature, and habitat (Moran 1999; Collin and Salazar 2010). Predation is often identified as the most influential factor on offspring survival in sessile organisms (Spight 1976). Although offspring size has been suggested to have a strong influence on the resistance of juveniles to predation (Rivest 1983; Barbeau and Scheibling 1994), evidence to the contrary has also been obtained (Gosselin and Rehak 2007). It emerges that the relationship between size and performance of juveniles under different types of predation pressure is incompletely understood in benthic species.

Here, we focus on taxa (unitary cnidarians) and metrics that have been understudied in the context of offspring size variations and performance to provide new empirical data on size effects. Experimental trials were conducted to gain a better understanding of the influence of size on the performance of premetamorphic (larva) and postmetamorphic (juvenile) stages in the brooding sea anemone *Urticina felina* (Linnaeus, 1761), which releases lecithotrophic (nonfeeding) larvae of various sizes (Mercier et al. 2011). Our specific aims were to (1) verify the effects of size on behavior, time to settlement, and survival of larvae, (2) compare lipid composition in larvae of different sizes, and (3) test size-related survival of juveniles in the presence of different sizes of their specialized predator. To test whether the size-related survival of juveniles varies between species, predation trials were also conducted on the juveniles of the sympatric live-bearing sea anemone *Aulactinia stella* (Verrill, 1864). This study presents evidence that the strength and direction of the relationship between offspring size and performance can vary species specifically, ontogenetically, and contextually.

## Materials and Methods

### Time to settlement and survival of small and large larvae of *Urticina felina*

Adults of *Urticina felina* were collected at a depth of ∽10 m off the Avalon Peninsula (Newfoundland, Canada) in June 2010 and were distributed into several holding tanks (20–40 L) supplied with unfiltered running seawater, at temperatures that followed the ambient annual cycle (0–10°C), under natural photoperiod. To compare the behavior of various-sized larvae from the same brood, four brooding females (41.2 to 212.9 g drained weight, with visible embryos/larvae) were maintained individually during the larval release period (July to September 2010). Lecithotrophic larvae were emitted through the mouth of the females and were collected within 24 h postrelease.

**Figure 1:**
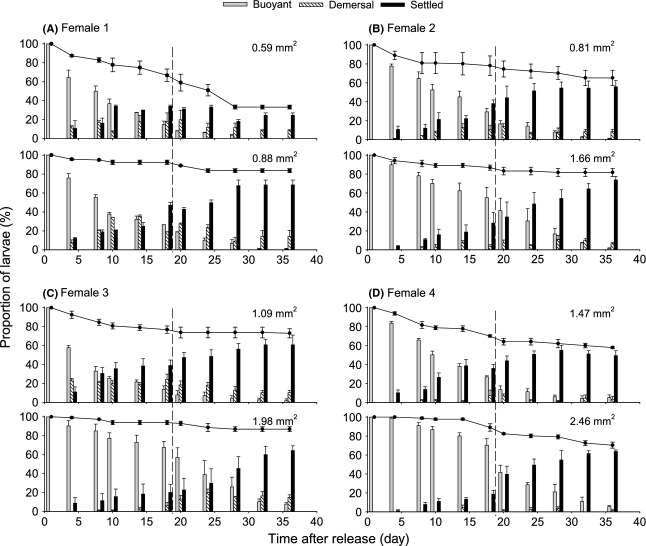
*Urticina felina*. Behavior (bars) and survival (line and associated data points) of small (upper panel) and large (lower panel) larvae released by four brooding females (A–D). Mean larval size for each group is indicated in the top right corner of each graph. Dashed lines indicate the introduction of the natural substratum on day 19. Data are expressed as mean ± SE (*n *=* *3 tanks for small and three tanks for large sibling larvae; 29–48 larvae per tank).

Between 191 and 277 larvae were collected from each of the four brooding females and used to test the influence of larval size on their performance (i.e., buoyancy, survival, and time to settlement). Larvae from the same brood (siblings) were examined under a Nikon SMZ1500 stereomicroscope (Nikon Canada Inc., Mississauga, ON, Canada) and then classified into two classes (small and large) based on their surface area. The mean size of small larvae was between 48.8 and 67.0% of the size of large sibling larvae, yielding significant size differences in each of the broods (Mann–Whitney or *t*-tests, *P* < 0.001) as illustrated in Fig.1.

Preliminary trials consistently showed that, regardless of size, the proportion of buoyant larvae dropped to 50% at 10 days postrelease when a rock (∽4 cm^2^) covered with coralline algae (*Clathromorphum* sp.) was offered (i.e., optimal substratum for settlement), whereas it dropped to 50% at 18 days postrelease in bare containers (mimicking suboptimal settlement conditions). Thus, the experiment was divided into two segments to test the influence of larval size (1) on the behavior under suboptimal settlement conditions (without preferred substratum), and (2) on the behavior of larvae when the optimal substratum became available (by exposing the same larvae to this new condition). Day 18 was chosen as the midpoint for the settlement experiment as per results described above.

Groups of small and large sibling larvae (*n *=* *29–48 per group; three groups for each size class in each female) were randomly distributed into six separate flow-through plastic tanks (2 L). The tanks were supplied with unfiltered running seawater (∽1.5 L min^−1^) and subjected to naturally fluctuating temperature and photoperiod (as described for adults). During the first experimental segment (days 1 to 18), containers were monitored every 2–4 days and larvae scored as: (1) buoyant (floating at the surface); (2) demersal, when larvae were on the bottom, but did not settle firmly; (3) settled, when they were firmly attached to the bottom or the sides of the container and could not be removed using a gentle jet of water. Survival rates were also recorded, as the percent number of offspring remaining (in all categories) at a given time relative to the initial number of larvae.

The second experimental segment (days 19 to 36) was performed to test the influence of larva size on behavior upon encounter with an appropriate settlement substrate (coralline algae added on day 19). The proportion of larvae in different categories and survival rates was still recorded every 2–4 days. Categories “buoyant” and “demersal” remained the same as in the first experimental segment, but the category “settled” then included larvae settled on bare and natural substrata. The experiment was terminated on day 36 when no or almost no buoyant larvae were left.

### Lipids in small and large larvae of *Urticina felina*

Larvae of *Urticina felina* were collected at the surface of the water column within 24 h postrelease for lipid analysis. Six samples of small and large sibling larvae (12–15 larvae per sample) were collected from three separate broods, measured and placed in 2 mL chloroform under nitrogen at −20°C for lipid analysis. In the determination of lipid concentration (*μ*g mm^−3^), the mean volume of small larvae from the three brooding females varied from 0.23 to 0.38 mm^3^ and that of large larvae varied from 0.44 to 0.98 mm^3^.

Extraction and analysis of lipids were based on standard methods for aquatic samples (Parrish 1999). Total lipids were extracted with a mixture of chloroform and methanol 2:1 (v:v). Lipid classes were determined using thin-layer chromatography with flame ionization detection (TLC/FID) with a MARK V Iatroscan (Iatron Laboratories, Tokyo, Japan). Lipids were separated in a three-stage development system. The first separation consisted of 25-min and 20-min developments in 99:1:0.05 hexane: diethyl ether: formic acid. The second separation consisted of a 40-min development in 79:20:1 hexane: diethyl ether: formic acid. The last separation consisted of 15-min developments in 100% acetone followed by 10-min developments in 5:4:1 chloroform: methanol: chloroform-extracted water. After each separation, the rods were scanned and the data were processed using the PeakSimple Chromatography software (V3.88, SRI Instruments, Torrance, CA, USA).

### Size-related survival of juveniles in the presence of predators

The nudibranch *Aeolidia papillosa* (Linnaeus, 1761) is a specialized predator of a number of sea anemones (Hall and Todd 1986), including *Urticina felina* and *Aulactinia stella* (Greenwood et al. 2004). Preliminary experiments showed that *A. papillosa* of all sizes could quickly feed on small individuals of *U. felina* and *A. stella* (within 30 min of contact) and that small specimens of nudibranchs (subadults) that ingested juveniles of both sea anemone species were ready to feed again after ∽24 h.

Adults (*n *=* *10; 3.8–19.3 g wet weight) and subadults (*n *=* *15; 0.02–0.6 g) of *A. papillosa* were collected at a depth of ∽10 m between May 2010 and January 2011 in Admirals Cove, Newfoundland, eastern Canada. The two categories of predators were used to determine how efficient and selective they were in the presence of small and large juveniles of *U. felina* (15-month old, Table1).

**Table 1:** Predation rates (%) and time (h) before feeding (mean ± SE; *n *=* *19–39) on juveniles of various sizes by the nudibranch *Aeolidia papillosa* within a 7-h experimental period. Data are shown for juveniles of two brooding sea anemones, *Urticina felina* and *Aulactinia stella*.

Predator *Aeolidia papillosa*	*Urticina felina* juveniles					*Aulactinia stella* juveniles				
	Small (2.2 ± 0.1 mg)		Large (12.0 ± 1.0 mg)		No predation	Small (6.4 ± 0.3 mg)		Large (78.5 ± 3.0 mg)		No predation
	Proportion (%)	Time (h)	Proportion (%)	Time (h)	Proportion (%)	Proportion (%)	Time (h)	Proportion (%)	Time (h)	Proportion (%)
Adults	0	–	0	–	100	15.8	1.8 ± 0.6	84.2	2.0 ± 0.2	0
Subadults	25.6	3.2 ± 0.5	48.2	4.2 ± 0.5	26.2	25.0	3.1 ± 0.8	39.3	3.5 ± 0.4	35.7

In *U. felina*, the size of settlers is well correlated with size of larvae at release (Sun et al. 2012). The experimental trial consisted of one *A. papillosa* offered simultaneously one small and one large juvenile sea anemone as potential prey. The trials were performed in round containers (21 cm in diameter) kept individually in 20-L flow-through tanks, supplied with a gentle flow (∽0.8 L min^−1^) ensuring uniform exchange and current of water through four equally spaced 3-cm meshed holes (500 *μ*m). Juveniles of *U. felina* were sorted and weighed (Table1), then allowed to recuperate for 24 h before the experiment. Sixty-four trials (39 and 25 replicates for subadult and adult predators, respectively) were performed between December 2010 and January 2011. Three to five trials were run simultaneously, and new *U. felina* juveniles were used as prey in each trial. To make sure that the predators were hungry, the interval between each replicate run was a minimum of 3 days (as per preliminary results above). At the onset of the trial, the predator was haphazardly introduced into the experimental container and left to acclimate for 1 h. Then, one small and one large juvenile sea anemone (sizes described above) were introduced simultaneously and placed at equal distance and angle from the predator. Predation was monitored every 30 min until a positive response (i.e., predator feeding on a prey or prey totally eaten by the predator) was scored, or up to 7 h, after which time the experiment was scored as null.

We also tested another species of sea anemone, *Aulactinia stella*, which is sympatric to *U. felina*. Adults of *A. stella* were collected at a depth of ∽10 m off the Avalon Peninsula (Newfoundland, Canada) from March to June 2010, and in January 2011, and maintained under the laboratory conditions mentioned previously for *U. felina*. Juveniles of *A. stella* were collected immediately after release and divided into two size classes (Table1). Forty-seven trials (28 and 19 replicates for subadult and adult *A. papillosa*, respectively) were performed between May and August 2010, and between December 2010 and January 2011. Experimental procedures were identical to the ones outlined above for *U. felina*.

### Data analysis

Repeated-measures analyses of variance (RM ANOVAs) were used to compare different variables in the performance of small and large sibling larvae of *Urticina felina* in two successive experimental segments (days 18 and 36). Relationships between mean larva size in a group and survival rates at the end of the two experimental segments were determined using Spearman's rank order correlation. Comparisons of different variables were also made between small and large *U. felina* larvae at the population level (i.e., across broods; where size categories were re-assigned across the full range of larvae, irrespective of parentage). Chi-square goodness-of-fit analysis was used to test whether predation rates on small and large juveniles were random.

Pearson's correlation was used to test the relationship between mean larva size and lipid content per larva (*μ*g ind^−1^). Comparisons of the proportions, and the amount (*μ*g ind^−1^) and concentration (*μ*g mm^−3^) of major lipid classes in small and large larvae of *Urticina felina* were made using *t*-tests at the population level and ANOVAs for within-brood analyses. Where assumptions of normality and equal variance failed, Mann–Whitney rank sum tests were used. The significance level for all tests was set at *P *<* *0.05. Data are expressed as mean ± SE.

## Results

### Behavior, time to settlement, and survival of *Urticina felina* larvae

**Figure 2:**
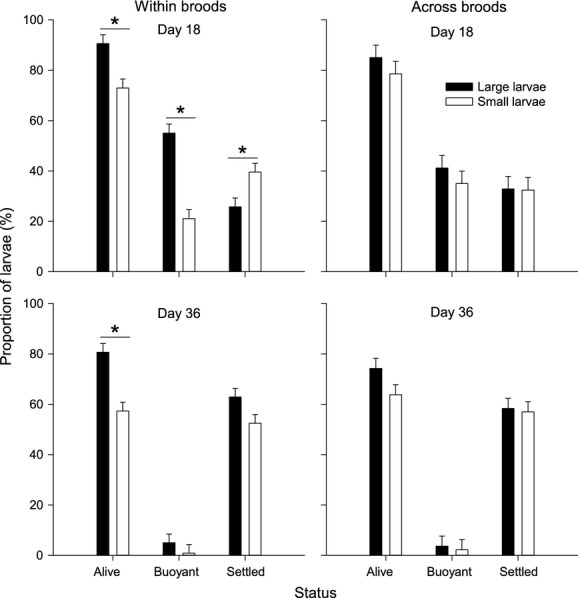
*Urticina felina*. Proportions of larvae that were alive, buoyant, and settled at day 18 (upper panels) and day 36 (lower panels), from analyses conducted within broods (left panels) or across broods, at the population level (right panels). Introduction of the natural substratum occurred on day 19. Data are expressed as mean ± SE (*n *=* *12, three replicates in each of four mothers). Asterisks show where significant differences occurred between large and small larvae (see text for Results).

While the size range of larvae differed among the four brooding females (i.e., the smaller larvae of some females were similar in size to the larger larvae of other females), comparable behavioral distinctions between large and small siblings occurred in all of the broods in the two experimental segments (Fig.1). However, results differed according to whether larvae size comparisons were made within or across broods (Fig.2).

According to a two-way RM ANOVA, both size (small or large) and time (day 18 or 36) had a significant effect on survival (*F*_1,22_ = 26.76 and 31.76, respectively, *P *<* *0.001), without any interaction (*F*_1,22_ = 1.61, *P *=* *0.218). Mean survival rates were lower among smaller larvae of a brood than among their larger siblings at day 18 (73.0 ± 3.1 vs. 90.6 ± 1.5%) and at day 36 (57.3 ± 4.9 vs. 80.7 ± 2.3%; Figs1 and 2). The time required for the proportion of buoyant larvae to drop to <50% was 11.5 ± 1.7 days in small larvae of a brood and 18.5 ± 3.5 days in large ones. There was a significant interaction between size and time on the proportions of buoyant larvae (*F*_1,22_ = 21.03, *P *<* *0.001) and settlers (*F*_1,22_ = 9.34, *P *=* *0.006). Independent analyses at the two time points revealed that the proportion of buoyant larvae was significantly lower in the smaller than larger siblings at day 18 (*P *<* *0.001, Fig.1) but not at day 36 (*P = *0.380), following the addition of the natural substratum. A different trend occurred in the proportion of settlers: Significantly more of the smaller siblings had settled at day 18 (*P = *0.017; Fig.1), whereas at day 36, the proportion of settlers was not significantly different between small and large larvae of a brood (*P *=* *0.068; Fig.2).

**Figure 3:**
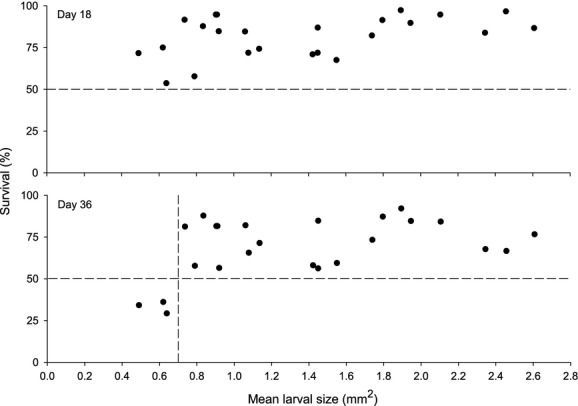
*Urticina felina*. Relationship between mean larval size (*n *=* *24 from four mothers) and survival at the end of the two experimental segments (upper panel at day 18, and lower panel at day 36). Horizontal dashed line indicates 50% survival rate, and vertical dashed line indicates 0.7 mm^2^ larval size.

To examine the influence of larval size on settlement at the population level (across broods), sizes were reclassified irrespective of parentage: All larvae measuring 0.59–1.14 mm^2^ were pooled into the small size class, and larvae between 1.42 and 2.61 mm^2^ were pooled into the large size class. Following this procedure, the mean size of small larvae was 0.84 ± 0.01 mm^2^ which represented 44.9% of the mean size of large larvae (1.87 ± 0.02 mm^2^). Analyses across broods yielded different trends compared to those previously obtained within broods (Fig.2). The RM ANOVA showed a significant effect of time (*F*_1,22_ = 30.62, *P *<* *0.001) but not size (*F*_1,22_ = 2.28, *P *=* *0.145) on survival. Mean survival rates did not significantly differ between the two size classes at day 18 (78.5 ± 3.9 vs. 85.0 ± 2.9%) or day 36 (63.7 ± 6.1 vs. 74.3 ± 3.6%). In addition, mean survival rate at day 18 was not correlated with mean larval size (Fig.3, *r*_*s*_ = 0.38, *n *=* *24, *P = *0.070); however, it was at day 36 (Fig.3, *r*_*s*_* *= 0.41, *n *=* *24, *P = *0.044). It is worth mentioning that survival rate after 36 days was 33.2 ± 2.0% when mean larval size in a group was <0.7 mm^2^, compared to 74.1 ± 2.6% when mean size was 1.48 ± 0.1 mm^2^ (Fig.3). The proportion of buoyant larvae decreased significantly over time (*F*_1,22_ = 60.46, *P *<* *0.001) but was never significantly different across the two size classes (*F*_1,22_ = 0.53, *P *=* *0.476; Fig.2). Similarly, time (*F*_1,22_ = 28.21, *P *<* *0.001) but not larval size (*F*_1,22_ = 0.06, *P *=* *0.815) had a significant effect on settlement rates at the population level.

### Lipid composition of *Urticina felina* larvae

**Figure 4:**
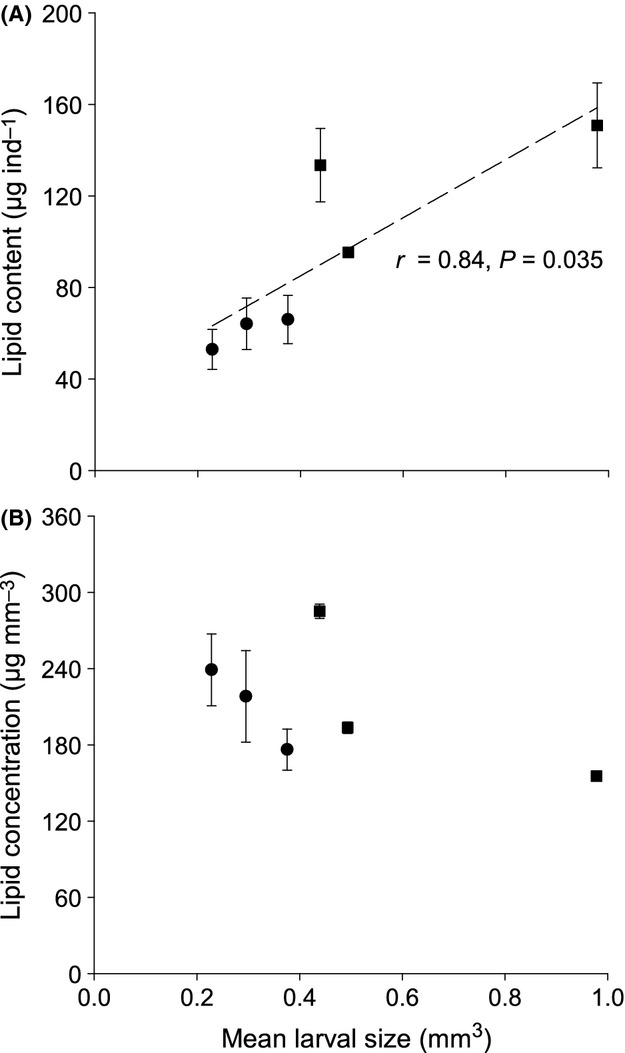
*Urticina felina*. (A) Significant linear relationship between total lipid content (*μ*g ind^−1^) and mean larval size. (B) Absence of relationship between lipid concentration (*μ*g mm^−3^) and mean larval size (*P *=* *0.237). Data are expressed as mean ± SE (*n *=* *6) for small (•) and large (▪) larvae.

Small and large larvae of *Urticina felina* were both composed of hydrocarbons (HC), wax esters/steryl esters (WE/SE), triacylglycerols (TG), free fatty acids (FFA), sterols (ST), acetone-mobile polar lipids (AMPL), and phospholipids (PL). At the population level, irrespective of parentage, total lipid content (*μ*g ind^−1^) was positively related to average larval size (*r *=* *0.84, *P = *0.035, *n *=* *6; Fig.4A). In contrast, lipid concentration (*μ*g mm^−3^) was not related to average larval size (*r *=* *−0.57, *P = *0.237, *n *=* *6; Fig.4B). Similarly, at the population level, total lipid content was significantly lower in small than in large larvae (*U *=* *0.00, *n*_small_ = 9, *n*_large_ = 9, *P* < 0.001), whereas lipid concentration was not (*U *=* *40.00, *P = *1.000). The amounts of most major lipid classes (*μ*g ind^−1^) were significantly lower in small than large larvae (Table2), except HC (*t*_16_ = 0.62, *P = *0.546). The proportions of all major lipid classes (=1% of total lipids) were similar in both small and large larvae (Sun et al. 2012). WE/SE was the most common lipid in both size classes, which comprised 53.5 ± 4.9% of total lipids in small and 58.6 ± 5.1% in large larvae.

**Table 2:** Mean lipid content (*μ*g ind^−1^) of major lipid classes (=1% of total lipids) in small and large larvae of the sea anemone *Urticina felina*.

Lipids	Small larvae	Large larvae
Hydrocarbons (HC)	2.65 ± 0.56^a^	3.03 ± 0.28^a^
Wax and steryl esters (WE/SE)	37.32 ± 3.95^a^	67.59 ± 7.85^b^
Free fatty acids (FFA)	0.42 ± 0.12^a^	2.82 ± 0.91^b^
Sterols (ST)	1.53 ± 0.18^a^	3.99 ± 0.87^b^
Acetone-mobile polar lipids (AMPL)	4.22 ± 1.75^a^	14.66 ± 4.36^b^
Phospholipids (PL)	14.86 ± 3.45^a^	34.07 ± 8.62^b^

Data are expressed as mean ± SE (*n *=* *9). Values with different superscript letters are significantly different (*t*-tests, *P *<* *0.05).

A closer within-brood examination showed that total lipid content was significantly lower in small than in large sibling larvae of a brood (*F*_3,17_ = 15.99, *P *<* *0.001), due to the significantly lower amounts of WE/SE (*F*_3,17_ = 7.10, *P = *0.005) and PL (*F*_3,17_ = 3.78, *P = *0.041) in small siblings. The amounts of the remaining major lipid classes, including HC, FFA, ST, and AMPL, were similar in all larvae inside a brood. The proportions of major lipid classes were similar in both small and large siblings, except for the proportion of HC, which was significantly higher in large larvae of a brood (*F*_3,17_ = 4.08, *P = *0.033).

### Predation on juvenile sea anemones of different sizes

Juvenile *U. felina* of all sizes were only susceptible to predation by subadult nudibranchs, as none of the adult nudibranchs fed on them within the experimental period, whereas 73.8% of subadult nudibranchs did (Table1). Among the latter, more fed on the larger prey offered. Specifically, 25.6% of subadult nudibranchs consumed the smaller *U. felina* juvenile, whereas 48.2% consumed the larger juvenile (Table1). These proportions differ significantly from an equal distribution, whether null trials are considered (*χ*^2^ = 9.95, df = 2, *P = *0.007) or not (*χ*^2^ = 6.92, df* *= 1, *P = *0.009). The average time before feeding by subadult nudibranchs was 3.2 ± 0.5 h on small *U. felina* juveniles, and 4.2 ± 0.5 h on large ones, with no significant difference (*U *=* *62.50, *n*_small_ = 10, *n*_large_ = 19, *P = *0.139).

In contrast to *U. felina*,*A. stella* juveniles, especially large ones, were more severely preyed upon by adults than by subadults of the nudibranch. All adult nudibranchs tested (100%) fed within the experimental period, compared to only 64.3% of subadult nudibranchs (Table1). Overall, 39.3% of subadult nudibranchs fed on small juvenile *A. stella* with a mean time before feeding of 3.1 ± 0.8 h, whereas 25.0% fed on larger juveniles within 3.5 ± 0.4 h. There was no significant departure from random distributions whether null trials were included (*χ*^2^ = 3.32, df = 2, *P = *0.190) or not (*χ*^2^ = 3.18, df = 1, *P = *0.075) and no difference in time to feeding (*t*_16_ = 0.51, *P = *0.615). On the other hand, larger *A. stella* juveniles were more susceptible than small ones to predation by adult nudibranchs. A significantly greater proportion of adult nudibranchs fed on large than on small *A. stella* juveniles (84.2 vs. 15.8%; *χ*^2^ = 46.79, df = 1, *P *<* *0.001; Table1) with similar mean delays before feeding of 2.0 ± 0.2 h vs. 1.8 ± 0.6 h (*t*_17_ = 0.27, *P = *0.792).

## Discussion

The present work provides new experimental results (summarized in Table3) in support of the assumption that offspring size influences premetamorphic as well as postmetamorphic performance, following slightly different schemes than previously shown in benthic marine organisms (Marshall and Keough 2003; Allen et al. 2008; Jacobs and Sherrard 2010). In the sea anemone *Urticina felina*, smaller larvae of a brood had lower survival than larger siblings and exhibited an inverse trend in the proportion of buoyant larvae and settlers, suggesting that smaller larvae settled more rapidly under suboptimal conditions, as per the “desperate larva” hypothesis. In contrast, the settlement of larger siblings was apparently driven by the presence of optimal substratum. A lipid analysis indicated that differences in survival and time before settlement in small and large sibling larvae may be due to the greater lipid content of the latter. The most abundant lipid class in all larvae was wax/steryl ester, which presumably provides larger larvae with more energy and prolonged buoyancy, enabling them to delay settlement until optimal conditions are encountered. Differences in survival and time before settlement were seen at the intrabrood versus across-brood population levels, suggesting that the relationship between larval size and performance is mediated by parentage. Finally, marginally different responses were evidenced when examining postmetamorphic competence in the form of susceptibility to predation in juveniles of *U. felina* (*<*12 mg) and those of a co-occurring sea anemone, *Aulactinia stella* (to 200 mg). Large juveniles of *U. felina* (originating from large larvae) were more susceptible than small ones against subadult predators, and all were ignored by adult predators. On the other hand, *A. stella* juveniles of all sizes were equally vulnerable to subadult predators, whereas larger juveniles were more vulnerable to adult predators.

**Table 3:** Summary of size-based performance of offspring at different ontogenetic stages in brooding sea anemones.

Species and life stage	Performance indicator	Small offspring	Large offspring
*Urticina felina*			
Larvae and new settlers, 18 day postrelease	Survival rate		
	Within brood	Lower	Higher
	Across broods	Similar	Similar
Larvae and settlers, 36 day postrelease	Survival rate		
	Within brood	Lower	Higher
	Across broods	Similar	Similar
Larvae, 18 day postrelease	Proportion of buoyant larvae		
	Within brood	Lower	Higher
	Across broods	Similar	Similar
Larvae, 36 day postrelease	Proportion of buoyant larvae		
	Within brood	Similar	Similar
	Across broods	Similar	Similar
Settlers, 18 day postrelease	Settlement rate (suboptimal conditions)		
	Within brood	Higher	Lower
	Across broods	Similar	Similar
Settlers, 36 day postrelease	Settlement rate (optimal substratum on day 19)		
	Within brood	Similar	Similar
	Across broods	Similar	Similar
Juveniles, 15–16 months	Predation by *Aeolidia papillosa*		
	Adults	Similar	Similar
	Subadults	Higher	Lower
*Aulactinia stella*			
Juveniles, 0–8 months	Predation by *Aeolidia papillosa*		
	Adults	Higher	Lower
	Subadults	Similar	Similar

### Offspring size and performance in premetamorphic stages

Survival enhanced by larger offspring size has been reported in colonial invertebrates, for example, bryozoans and ascidians (Marshall and Keough 2003, 2005) and corals (Isomura and Nishihira 2001). However, the relationship between offspring size and survival was suggested to vary with time, that is, the effects only persisting for a short period of time. For example, colonies of the ascidian *Diplosoma listerianum* that developed from larger larvae had larger feeding structures and higher survival than those developed from smaller larvae after 2 weeks, but not after 3 weeks in the field (Marshall and Keough 2005). Here, in contrast, larger larvae of the unitary species *U. felina* exhibited consistently greater survival than their smaller siblings after 18 and 36 days. When mean larval size in a group was <0.7 mm^2^, survival rates were always lower than 50%. *Urticina felina* larvae = 0.6 mm^2^ (coined mega-larvae) were shown to be formed by fusion of sibling embryos (Mercier et al. 2011; Sun et al. 2012). Greater survival rates in larger mega-larvae supports the adaptive role of fusion in creating longer-lived and more dispersive larvae in this species. However, survival rates were similar in large and small size classes at the population level (irrespective of parentage), which suggests that parental effects are acting on the offspring size–performance relationship and stresses the importance of conducting future studies at the within-brood/clutch level.

Behavioral differences during settlement have been reported in many benthic marine organisms (reviewed by Raimondi and Keough 1990). The latter authors suggested that larval behavior variability may be caused by “genetic variation among larvae, ontogenetic changes in behaviors, parental environmental effects, modification of response by other environmental cues, or the overriding of behavioral responses by physical process”. However, the relative contribution of genetic and environmental factors to larval behavior variability and the detailed mechanisms underlying this variability are still largely unknown. While the present study did not set out to examine this question, it seems to show that larval size in *U. felina* not only influenced the final results but also the dynamics of settlement. For example, proportions of buoyant individuals were lower in smaller than in larger siblings of a brood under suboptimal settlement conditions before the addition of the natural substratum. However, those proportions were not significantly different between the two size classes at the end of the experimental period (36 days). These trends suggest that smaller individuals need to settle more rapidly, but that final settlement rates remain similar in both size classes. Ultimately, size-related performance may therefore be mediated by the proximity of suitable settlement sites. Worth mentioning is the fact that the proportion of settled larvae was not significantly different between large and small larvae at any time at the population level, emphasizing the point made earlier about studies needing to consider intrabrood versus across-brood responses.

The influence of offspring size on settlement behavior (desperate larva theory) has been reported in marine invertebrates (Toonen and Pawlik 2001; Marshall and Keough 2003). For example, larger larvae of the bryozoan *Bugula neritina* had a more variable swimming period before settlement compared to smaller ones and, although small and large larvae were capable of settling, smaller larvae settled sooner than larger larvae (Marshall and Keough 2003). Similarly, a field study showed that the size of settlers in the bryozoan *Watersipora subtorquata* was larger on rough surfaces, compared to smooth plates, which suggested that smaller larvae were less selective for habitat (Marshall and Keough 2003). Based on our study, it is likely that the effects of larva size on swimming time could be levelled in the presence of a strong settlement inducer (optimal conditions) from the onset. However, the size-related variability in settlement behaviors among sibling larvae of *U. felina* may serve as a dispersal strategy, that is, to maintain recruitment of some offspring (smaller in size) closer to the parental habitat (philopatry), while allowing the larger ones to disperse more widely, particularly when incentives for settlement are weaker (e.g., suboptimal environment, competition, predation). In brooding species that release fully formed larvae within a short time, such as *U. felina*, this strategy may have evolved to decrease the intrinsic effects of competition among sibling settlers. Offspring size variation as a strategy to decrease intraspecies competition has been reported in other marine invertebrates. For instance, larger egg size corresponded to longer planktonic period in three lecithotrophic species, the ascidians *Phallusia obesa* and *Ciona intestinalis* and the echinoid *Heliocidaris erythrogramma*, suggesting that offspring from large eggs would disperse further than those from small eggs and that spreading of offspring may decrease intraspecific competition (Marshall and Bolton 2007).

### Larval size and lipid composition in *Urticina felina*

Offspring size, especially egg size, has been suggested to reflect parental investment per offspring and to be an indication of organic content in marine invertebrates (Jaeckle 1995). It has been shown that larval settlement behavior and dispersal patterns might be determined via lipid content, composition, and allocation (Harii et al. 2007) and that marine invertebrates with lecithotrophic (nonfeeding) larvae may mediate dispersal potential of their offspring by manipulating larval size, because, as mentioned above, small larvae tend to become less discriminating in their choice of settlement substrata.

Although larval size in *U. felina* does not reflect initial egg provisioning due to fusion among siblings (Mercier et al. 2011; Sun et al. 2012), the total lipid content per larva (*μ*g ind^−1^) followed the predicted increase with size. Further examination showed that the significantly lower lipid content in small than in large larvae within a brood was due to lower amounts of wax esters/steryl esters (WE/SE) and phospholipids (PL). WE/SE was the most abundant lipid class in both small and large larvae of *U. felina* comprising 54–61% of total lipids. In other members of the family Actiniidae, WE has comprised 35–40% of whole animal total lipid (Nevenzel 1970). WE is the major lipid considered to govern buoyancy and act as energy reserves in marine organisms (Lewis 1970; Nevenzel 1970); hence, changes in the proportion of WE could influence the position of larvae in the water column and control their dispersal. For example, Harii et al. (2007) found that the WE content changed significantly over time in the larvae of the hermatypic coral *Acropora tenuis* and suggested that WE might be an energy source for metamorphosis and settlement. Thus, we propose that the lower amount of total lipids and especially WE/SE in small larvae of *U. felina* explains why they stay buoyant for a shorter period than larger siblings under nonoptimal settlement conditions. It is worth mentioning that the total lipid content (*μ*g ind^−1^) in large larvae was solely due to scaling, as lipid concentration (*μ*g mm^−3^) was similar in all larvae. Studies on size-specific energy consumption are needed to confirm whether larger larvae have proportionally more energy reserves than smaller ones.

### Offspring size and postmetamorphic performance (as susceptibility to predation)

To date, more studies have focused on competition (conspecific densities; Allen et al. 2008) than predation as a biotic influence on postmetamorphic performance. Nevertheless, offspring size has been suggested to influence resistance to predation (Rivest 1983; Barbeau and Scheibling 1994). Smaller hatchlings of the neogastropod *Searlesia dira* were preferentially selected by smaller hermit crab predators, whereas larger crabs did not show any feeding preferences related to prey size (Rivest 1983). Furthermore, the general role of body size in predator–prey interactions has been shown in marine invertebrates, fishes, and insects (Juanes 1992; Lundvall et al. 1999; Berger et al. 2006). For invertebrate predators, prey vulnerability was predicted to initially increase with size to a maximum and decrease thereafter. This dome-shaped function has been suggested to be a combined effect of the predator's ability to detect small prey and its ability to capture large prey (Christensen 1996; Lundvall et al. 1999). Feeding preferences of a predator of a given size are determined by the combination of the energy intake efficiency (Stephens and Krebs 1986) and the cost of predation (Stephens and Krebs 1986; Juanes 1992). Smaller predators preferentially feeding on smaller prey have been reported (Juanes 1992; Barbeau and Scheibling 1994).

In the present study, *U. felina* juveniles, irrespective of their size, were more vulnerable to subadults of the nudibranch *Aeolidia papillosa*, as no adult nudibranchs fed on them. This is likely because large adult nudibranchs are less inclined to spend energy preying on such small prey as *U. felina* juveniles (<12 mg). Further support for this assumption is provided by the fact that large juveniles of *U. felina* were more frequently consumed by subadult nudibranchs than small ones. A different scenario was observed in interactions between nudibranchs and much larger prey, that is, juveniles of the sea anemone *Aulactinia stella* (to 200 mg), where subadult nudibranchs were less inclined to feed on the juveniles than adult nudibranchs. Under the predation of subadult nudibranchs, small and large *A. stella* juveniles suffered equal predation rates, whereas larger juveniles suffered higher predation rates than their small siblings when exposed to adult nudibranchs. The nudibranch *A. papillosa* uses mucus to counteract its prey's nematocysts (Greenwood et al. 2004), but it may still risk injury or death when the prey is large enough (Conklin and Mariscal 1977). Thus, the different feeding preference of nudibranchs on the *A. stella* juveniles of various sizes is possibly related to the higher risk of injury from the prey's nematocysts for small subadult nudibranchs than for the adults. In summary, the interaction between juvenile sea anemones and their specialized predator is driven both by the size of the prey and the size of the predator, in line with the general principles of the foraging theory (Stephens and Krebs 1986), generally placing larger offspring at a disadvantage.

### Implications

Taken together, our results indicate that the relationship between offspring size and performance is a difficult one to assess, being dependent on a complex suite of environmental and biotic factors encountered at different life stages: here, the availability of optimal substratum during settlement and the type of predation at the juvenile stage. Clearly, the general assumption that larger offspring perform better does not hold true in the present study, especially at the postmetamorphic stage. Challenges to this common assumption have previously been reported in terrestrial and aquatic vertebrates (Dibattista et al. 2007; Warner and Shine 2007; Maddox and Weatherhead 2008) and in ascidians (Jacobs and Sherrard 2010). While difficulties in interpreting maternal effects and costs/benefits across life stages have been emphasized (Marshall and Uller 2007; Allen and Marshall 2014), size is still commonly viewed as an indicator of offspring quality/fitness in defining strategies adopted by mothers. We show here, in line with a growing number of studies, that this underlying assumption is tenuous, further challenging the current framework under which maternal and offspring effects are examined. We conclude that the importance of offspring size may be overestimated relative to other traits in defining life-history strategies and that future studies should give more consideration to intraclutch (genetic) effects, ontogeny, and the interplay of different influential factors (e.g., competition and predation).
